# Supplementary material for: Transient stripping of subducting slabs controls periodic forearc uplift
Source: Nat Commun. 2020 Apr 14;11:1823. doi: 10.1038/s41467-020-15580-7 (PMC7156703; doi:10.1038/s41467-020-15580-7)
Supplement: Supplementary file 1 — Supplementary Information [file 41467_2020_15580_MOESM1_ESM.pdf]

Transient stripping of subducting slabs controls periodic forearc uplift

Armel Menant[1,2,*], Samuel Angiboust[1], Taras Gerya[3], Robin Lacassin[1], Martine Simoes[1], Raphael Grandin[1]

[1] *Université de Paris, Institut de physique du globe de Paris, CNRS, F-75005, Paris, France.*
[2] *Present address: GFZ Helmholtz Centre Potsdam, German Research Centre for Geosciences, Telegrafenberg, 14473, Potsdam, Germany.*
[3] *Institute of Geophysics, Swiss Federal Institute of Technology (ETH), Zürich, Switzerland.*

** Corresponding author: armel.menant@gfz-potsdam.de*

Content

- **Supplementary Note 1:** Tectonic underplating and forearc topography along the Circum-Pacific belt
- **Supplementary Note 2:** Resolution test
- **Supplementary Note 3:** Influence of plate convergence rate on tectonic underplating and associated topographic response
- **Supplementary Note 4:** Role of subducting sediment thickness on accretionary dynamics and forearc topography
- **Supplementary Figs. 1-7**
- **Supplementary Tables 1-2**
- **Supplementary References**

Supplementary Note 1: Tectonic underplating and forearc topography along the Circum-Pacific belt

Active convergent margins along the Circum-Pacific belt display a wide range of first-order subduction parameters including their thermal structure[1], upper- and lower-plate kinematics[2] and mass flux[3,4]. Tectonic underplating is interpreted from geophysical imaging below many forearc regions (Fig. 1a)[5–8] and is supported by the ubiquitous observation of paleo-accretionary duplexes attesting for past underplating events[9–13]. Here, we briefly review the forearc morphology and topography and associated recent vertical surface displacements along subduction segments where tectonic underplating is suspected (Fig. 1; see also Supplementary Fig. 1).

Southwest Japan

The forearc domain of the Nankai accretionary margin is characterized by a high coastal topography with an average and maximum elevation of ~450 m and ~1,590 m, respectively. It consists in a ~120-km-wide belt, which extends laterally from Honshu to Kyushu islands and forms the entire Shikoku island (Supplementary Fig. 1a). Landward, an inner-forearc depression forms the Inland Sea, which separates Shikoku from the active arc on Honshu. The depth of the subducting oceanic lithosphere below the high coastal topography is estimated to ~30-40 km, implying that it is located trenchward from the intersection of the downgoing slab with the continental Moho of the upper plate (i.e., ~40 km depth; see ref[3] for a compilation of the crustal thickness of the overriding continental plates). In addition to this first-order topographic pattern, a trench-parallel succession of smaller-scale topographic highs and lows is observed in the outer-forearc domain[14] (Supplementary Fig. 1a). The antiformal-shaped coastal promontories (i.e., Ashizurri, Muroto and Kii) experienced differential uplift in the middle-late Miocene as revealed by apatite fission track ages[15,16], while the timing for the formation of offshore basins (i.e., Hyuga, Tosa, Muroto, Shima and Enshu) remains poorly constrained, except for the Pliocene-Quaternary Kumano basin[17,18]. Despite the lack of robust constraints on the basins, these topographic variations strongly suggest recent trench-parallel differences in forearc uplift and subsidence along the Nankai margin.

Alaska

The Alaska accretionary margin displays forearc topographic highs mostly represented by the Kodiak island and the Kenai peninsula, where the elevation reaches up to ~1,000-m high. (Fig. 1b; see also Supplementary Fig. 1b). Another structural high is recognized west of Kodiak

and corresponds to the Semidi islands. Kodiak consists in a Jurassic-Eocene paleo-accretionary complex forming a trench-parallel antiform, which experienced two rapid cooling and exhumation events in the latest Cretaceous-early Tertiary and Eocene-Oligocene[12,19]. Actively subsiding areas are observed in between these topographic highs, including the late Miocene-Quaternary Tugidak basin where compressional and extensional deformations are recorded in the trenchward and landward side of the basin, respectively[20]. This trench-parallel succession of topographic highs and lows is directly located above a ~30-km-depth plate interface, trenchward from the intersection with the continental Moho (i.e., ~36 km depth). Landward, the Shelikof Strait and Cook Inlet form a topographic depression, which marks the transition towards the arc domain.

Cascadia

The forearc domain along the Cascadian accretional subduction zone is marked by a high coastal topographic belt, which extends from the Insular Range of Vancouver island to the Coast Range in Washington and Oregon States (Supplementary Fig. 1c). This belt has a ~560-m-high average topography with a maximum elevation at ~2,240-m high (Fig. 1b) and is separated from the arc domain by a forearc depression extending from the Georgia Strait to the Puget-Willamette Lowlands. The Olympic Mountains belong to the Coast Range and consist in a late Oligocene-early Miocene paleo-accretionary wedge, which has been exhumed since the middle Miocene. Trenchward, a series of late Cenozoic offshore basins are recognized in seismic profiles (e.g., Olympic, Wilapa, Astoria and Newport basins), recording a protracted subsidence history interrupted by a late Miocene uplift event[14,21]. Directly below the high forearc topography, the plate interface is located at depth of ~30-40 km, in front of the intersection with the continental Moho (i.e., ~45 km depth).

North Chile

The North Chilean erosive margin (~19-27 °S) is characterized by a relatively continuous high forearc topography called the Coastal Cordillera (Supplementary Fig. 1d). It displays an average and maximum elevation of ~1,350 m and ~2,720 m, respectively (Fig. 1b) and is located at ~40-50 km above the intersection of the plate interface with the continental Moho (i.e., ~45 km depth). Landward, the topography decreases to form the Central Depression (or Longitudinal Valley), which extends in front of the Main Cordillera and marks the transition towards the arc domain. Timing for rising of the Coastal Cordillera remains controversial but an overall uplift of the forearc domain is inferred at least since the early Miocene[22,23],

interrupted by periods of quiescence or slow subsidence[24,25]. The latter are mostly deduced by the stratigraphic record of several half-graben basins recognized along the Coastal Cordillera and partly extending offshore, which suggest a middle-late Miocene-Pliocene slow subsidence followed by an uplift event through the Pleistocene[24,26] but with possible variations in the timing along the margin. These basins are particularly well exposed on the Mejillones peninsula, which forms a prominent coastal promontory characterized by normal faulting, antiformal bending and subaerial emergence since the Pleistocene[27,28].

Central Chile

The forearc domain of the accretional Central Chilean margin (~35-44 °S) is marked by a high topography along the coastline, corresponding to the southern extension of the Coastal Cordillera but with lower elevations (i.e., ~180-m high in average and a ~1,100-m-high maximum altitude; Fig. 1b; see also Supplementary Fig. 1e). Directly below, the plate interface is located at ~40 km depth, in front of the intersection with continental Moho (i.e., ~45 km depth). Landward, the Central Depression separates the Coastal Cordillera from the Main Cordillera and the arc domain. Tectonostratigraphic studies seem to reveal regional variations in vertical surface displacements of the forearc domains, with long-lived basins developed west of the Coastal Cordillera, which experienced a middle Miocene-early Pliocene subsidence before being inverted and uplifted since the late Pliocene[29,30]. This subsequent uplift event led to the partial emergence of these forearc basins, notably on the Arauco peninsula, which forms a dome-like structure exhumed since the Pliocene[31].

Supplementary Note 2: Resolution test

To test the effect of numerical resolution on the accuracy of our results, an additional model has been performed from the reference experiment but with a lower spatial resolution for the Eulerian grid in the vicinity of the plate boundary; i.e., 1.5 x 1.0 km (instead of 0.5 x 0.5 km) in x and y, respectively. Indeed, ref[32] observed that calculated surface topography using the sticky-air method may be significantly overestimated in geodynamic models with low spatial resolution.

Results from the resolution test are consistent with the reference experiment (compare Supplementary Fig. 3 and Figs. 2 and 3), as they are characterized by:

- a steady-state subduction history where both frontal and basal accretion are predicted over a >75 Myr-long period,
- a 50-60-km-wide frontal sedimentary wedge,
- a duplex structure made of sedimentary and basaltic slices regularly underplated between ~18-28-km depth and subsequently exhumed up to the surface following an overall vertical mass flow,
- a long-term fore-arc topography characterized by a ~8,000-m-deep trench and a >1,000-m-high coastal topography composed of two highs and separated from the arc region by an inner-forearc depression,
- a periodic growth of the large, positive coastal topography by an alternation of uplift and subsidence events correlated to transient underplating events and subsequent wedge re-equilibration, respectively.

It is worth noting that the topographic signal displays a robust periodicity of 3.1 ± 0.3 Myr, which is similar to the high-resolution, reference experiment (i.e., 2.7 ± 0.4 Myr). This resolution test thus evidences that the periodic underplating events and resulting forearc topographic evolution predicted in this study are robust, independently from the spatial, numerical resolution.

Supplementary Note 3: Influence of plate convergence rate on tectonic underplating and associated topographic response

Plate convergence rate has a first-order control on the thermal structure, stress loading and mass flux along subduction zones. To evaluate the role of this crucial parameter, we performed a series of numerical experiments by prescribing different plate convergence rates (V_{conv} comprised between 2 and 10 cm yr^{-1}; Supplementary Table 1, Supplementary Figs. 4 and 5 and Supplementary Movies 2 and 3).

Results from the fast-subduction model

Our numerical experiment displaying a fast convergence rate (i.e., V_{conv} = 8 cm yr^{-1}; model sed8.0) shows the rapid development of a thick duplex by successive underplating events of mostly sedimentary material (Supplementary Fig. 4a). Several basaltic tectonic slices are also formed at ~20-30-km depth but few of them are preserved in the final duplex structure. Driven by an overall vertical mass flow, the progressive exhumation of the duplex up to the surface is achieved by surface erosion and normal faulting (Supplementary Fig. 4b). Resulting long-term forearc topography is characterized by a ~2,000-m-high coastal topography composed of two highs, which locates directly above the basal accretion sites and a deep inner-forearc depression extending above the mantle wedge (Supplementary Fig. 4b). The temporal evolution of the forearc topography is controlled by a succession of uplift and subsidence events, which controls the long-term rise of the coastal topography before it reaches an equilibrium at ~2,000-m high (Supplementary Figs. 4c and 4d). These topographic oscillations are characterized by an uplift and subsidence rate varying between ~0.5 and ~2 mm yr^{-1} and a periodicity of ~1.2 Myr according to a Fourier transform calculation (Supplementary Figs. 4e and 6).

Results from the slow-subduction model

By prescribing a slow plate convergence rate (i.e., V_{conv} = 2 cm yr^{-1}; model sed2.0), our model predicts that tectonic underplating takes place by a mostly horizontal mass flow at the base of the forearc crust, contributing to the formation of a ~100-km-thick accretionary wedge (Supplementary Figs. 5a and 5b). The latter is largely composed of basaltic slices. These slices are continuously stripped from the subducting oceanic crust to the base of the forearc domain, which experiences a compressional deformation with thrust faulting. Resulting topography is extremely low (i.e., <1,000 m below the sea level) and shows non-periodic, slow vertical

variations (Supplementary Figs. 5c-5e and 6). These Myr-scale topographic fluctuations are mostly controlled by the long-term dynamics of the subducting lithosphere with, for instance, slab flattening at ~35-40 Myr, which results in a transient decrease of the forearc topography (Supplementary Movie 3).

Plate convergence, vertical vs. horizontal mass flux and topographic variations

Results from our numerical experiments evidence the major role of plate convergence rate on the accretion dynamics and topographic evolution of subduction zones. It appears that the nature of accreted material is partly controlled by the material flux entering the trench. Indeed, fast subduction provides high-enough sediments to be accreted at the base of the forearc domain without involving the underlying oceanic crust. The long-term consequence of this fast dynamics is the formation of a mostly sedimentary duplex (Supplementary Fig. 4). Alternatively, slow subduction and associated low material influx results in the stripping of the basaltic crust besides the overlying sediments, resulting in an accretionary wedge largely composed of dense oceanic material (Supplementary Fig. 5).

A first, critical consequence of the different composition of the accretionary wedge is its buoyancy. A buoyant sedimentary duplex promotes its own exhumation (sustained by ongoing underplating from below) and results in an overall vertical flow throughout the forearc domain, supporting a high coastal topography (Fig. 2; see also Supplementary Fig. 4). Alternatively, a less-buoyant mafic-dominated complex favours a plate-motion-driven, horizontal flow preventing such a high topography (Supplementary Fig. 5).

The second major implication is the modification of the Myr-scale topographic signal because the nature of underplated material controls the duration of the tectonic slicing events along with the periodicity of the topographic oscillations. Accordingly, only-sediment underplating events are closer in time than sediment-and-basalt underplating events, which results in a decreasing periodicity of topographic variations when increasing the plate convergence rate and the amount of sediments accreted at depth (Fig. 4; see also Supplementary Fig. 6). Note that a fast convergence rate allows for a more rapid stress build-up along the plate interface, which also contributes to decrease the periodicity of slicing events and associated topographic oscillations (see details in the main text). For slow subduction (i.e., $V_{conv} < 4$ cm yr^{-1}), however, tectonic underplating is achieved by a mostly horizontal mass flow, which prevents any significant vertical topographic signal.

Supplementary Note 4: Role of subducting sediment thickness on accretionary dynamics and forearc topography

Another critical parameter controlling the mass flux in subduction zones is the amount of sediments entering the trench. To evaluate the importance of this parameter, we performed an additional experiment with no pelagic sediments on top of the oceanic crust (model nosed5.0; Supplementary Fig. 7; Supplementary Movie 4). In this experiment, the initiation of the subduction zone is associated with tectonic underplating of a thick tectonic slice of basaltic crust, similarly to our reference model. Shortly after, the subduction regime becomes erosive, characterized by trench retreat, minor frontal accretion (resulting in a 20-30-km-wide frontal wedge) and basal erosion of initially underplated basaltic material and continental crust (Supplementary Fig. 7a). Because of the overall subsidence of the forearc margin, the predicted topography is significantly low with a ~8,000-m-deep trench and a maximum topographic high of ~500-1,000 m below the sea level (Supplementary Fig. 7b). The evolution of the forearc topography in this experiment evidences no periodicity in the vertical variations at the surface, which are, instead, controlled by transient mass fluxes and erosive dynamics along the margin (Supplementary Figs. 7c-7e). Rapid subsidence thus occurs at ~46-47 Myr as a result of a sudden basal erosion event, while the subsequent fast uplift is controlled by an increasing amount of subducting material (mainly coming from the erosion of the frontal wedge; Supplementary Movie 4).

Results from this numerical experiment first support the crucial role of tectonic underplating (absent here) on the Myr-scale topographic signal predicted in most of other simulations. In addition, it provides insightful constraints on the forearc topography for a long-lived, steady-state erosive margin. By comparing these results with the North Chilean erosive margin, it is noteworthy that the higher forearc elevation and the total tectonic relief (i.e., from the trench to the forearc high) are much lower in our experiment (compare Fig. 1b and Supplementary Fig. 7b), supporting the idea that other tectonic processes, such as coeval tectonic underplating, have to be considered to explain such a high coastal topography[24,33].

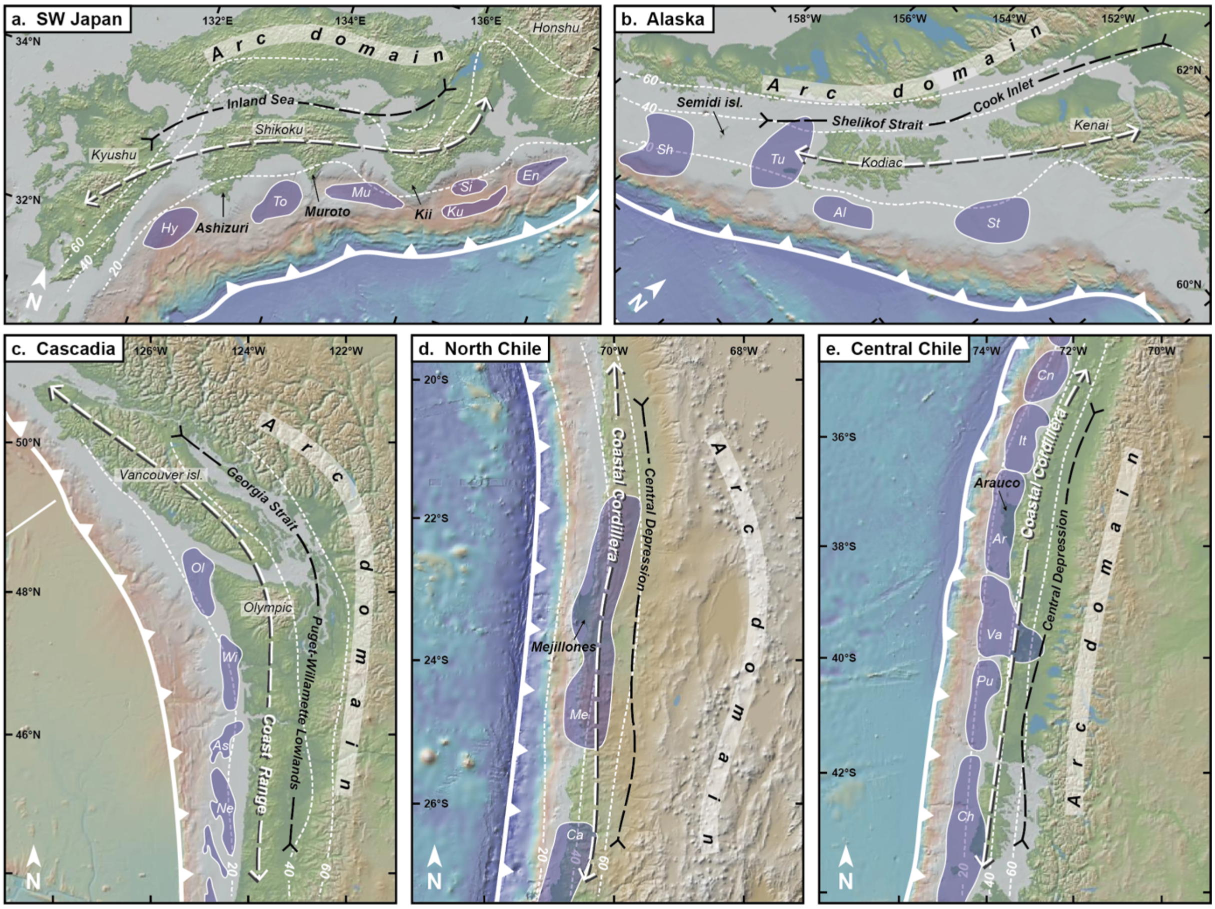

Supplementary Fig. 1 – Surface morphology of forearc domains along the Circum-Pacific belt where tectonic underplating is suspected from geophysical imaging. a-e. A similar forearc topographic pattern is recognized all along the SW-Japan, Alaska, Cascadia, North and Central Chile margins (see also Fig. 1). It consists in a trench-parallel high coastal range (dashed thick white line) followed by a depression (dashed thick black line), which marks the transition towards the arc domain. The 20-, 40- and 60-km isodepths of plate interface are depicted as dashed thin lines, according to ref[34], except for SW Japan[35]. Recent outer-forearc basins are represented as blue patches, for SW Japan and Cascadia[14], Alaska[20], North Chile[24] and Central Chile[30]. For-arc basins, *Hy*: Hyuga, *To*: Tosa, *Mu*: Muroto, *Si*: Shima, *Ku*: Kumano, *En*: Enshu, *Sh*: Shumagin, *Tu*: Tugidak, *Al*: Albatross, *St*: Stevenson, *Ol*: Olympic, *Wi*: Willapa, *As*: Astoria, *Ne*: Newport, *Me*: Mejillones, *Ca*: Caldera, *Cn*: Chanco, *It*: Itata, *Ar*: Arauco, *Va*: Valdivia, *Pu*: Pucatrihue, *Ch*: Chiloé. See Supplementary Note 1 for details. Maps have been extracted from the Global Multi-Resolution Topography (GMRT) synthesis[36] with GeoMapApp [www.geomapapp.org].

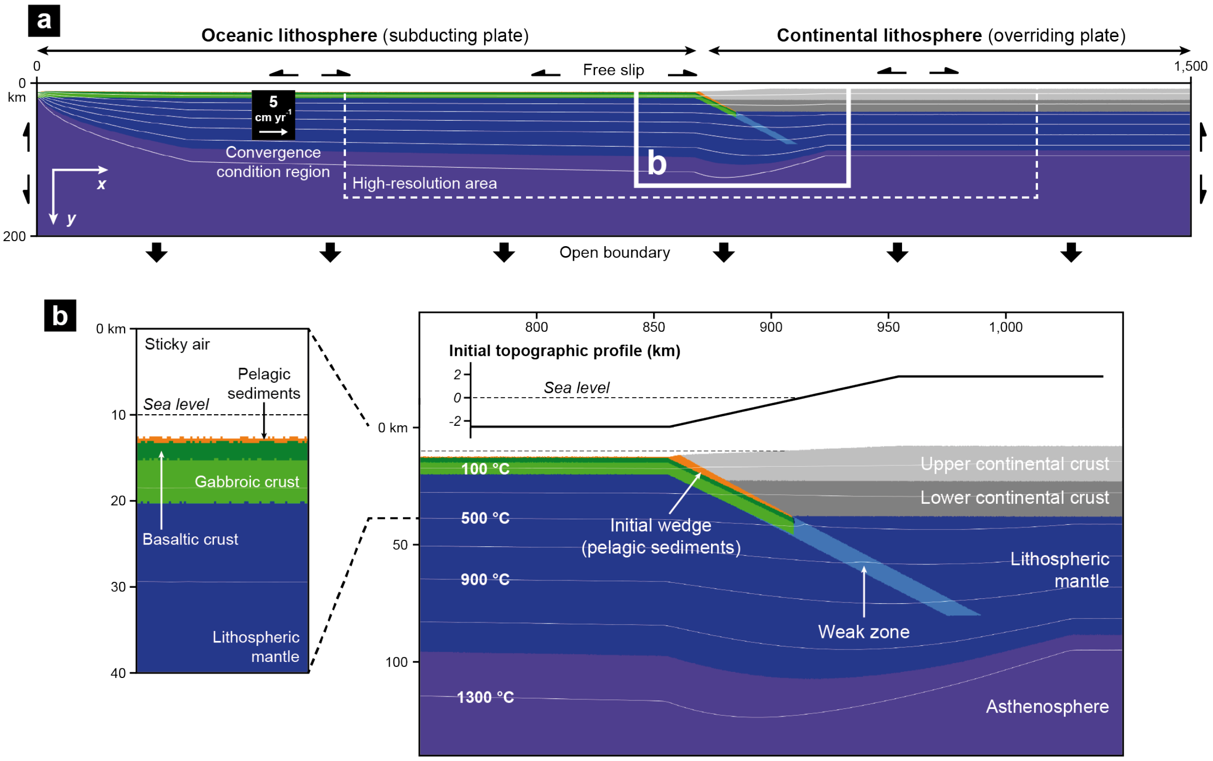

Supplementary Fig. 2 – Numerical setup of ocean-continent subduction zone. a. Compositional map of the whole computational domain. **b.** Zoom of the compositional map on the initial plate boundary. Details on the layering of the oceanic crust is shown as inset. Associated line graph shows the initial topographic profile at the plate boundary with a 2.5 vertical exaggeration. Note that y coordinates on the compositional maps are defined from the top of computational domain (i.e., $y = 0$ km), while the vertical scale of the topographic profile is defined from the sea level (i.e., $y = 10$ km). For further details on the numerical setup, the reader is referred to the Methods section.

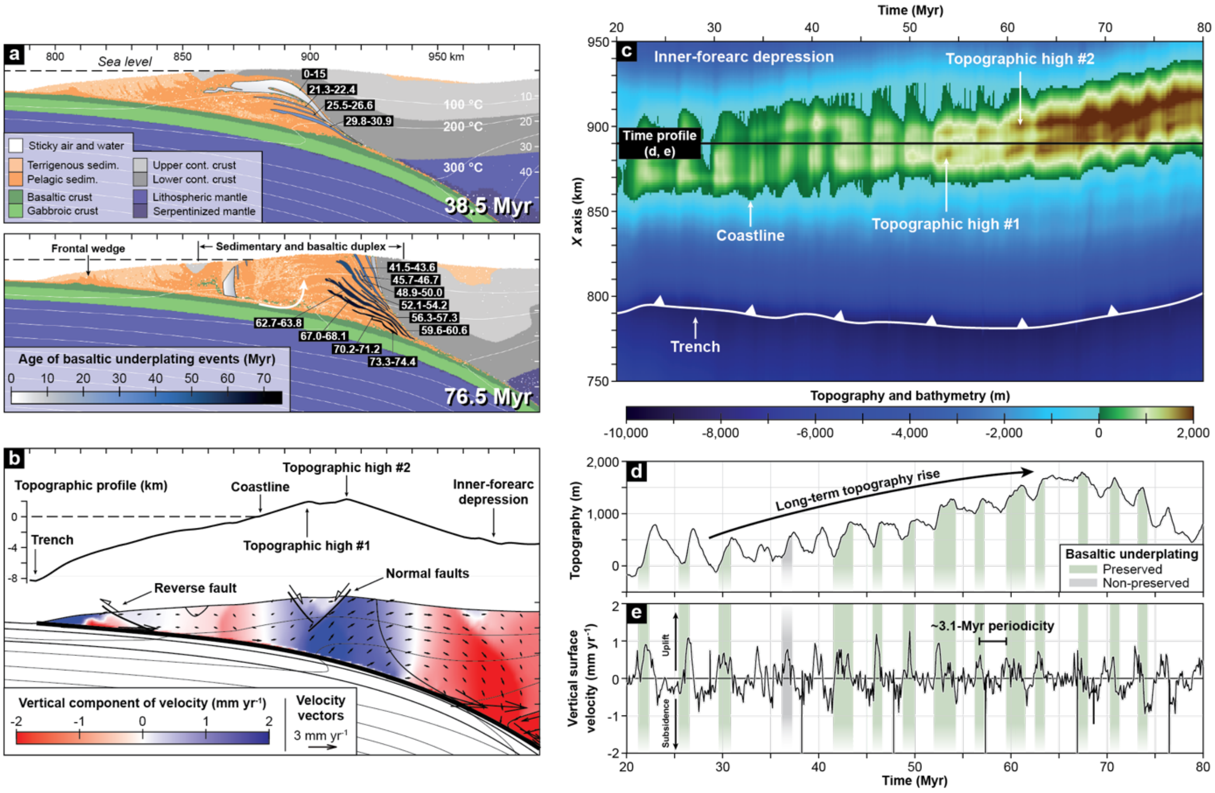

Supplementary Fig. 3 – Resolution test from the reference experiment but with a lower spatial resolution for the Eulerian grid (i.e., 1.5 x 1.0 km in *x* and *y*, respectively). a. Compositional maps focusing on the forearc domain where tectonic underplating of sedimentary and basaltic material is achieved by an overall vertical flow (thick white arrow). Once inserted in the duplex, the basaltic slices are highlighted with white-to-blue colours depending on the timing of basal accretion (black boxes; time in Myr). **b.** Velocity map (vertical component only) of the forearc domain during a discrete underplating event. Red and blue colours represent downward and upward vertical displacements, respectively. Black arrows depict the calculated global velocity field. The modelled topographic profile across the forearc domain is depicted with a 2.5 vertical exaggeration. **c.** Dynamics of forearc topography showing the periodic evolution of the coastal topography with alternating uplift and subsidence events and a horizontal migration of the coastline. **d, e.** Time profiles showing the topography (**d**) and vertical surface velocity (**e**) variations of the coastal topography from 20 to 80 Myr (see (**c**) for the exact location of the profile). Green (or grey) bands denote the timing of tectonic underplating of basaltic slices, which are preserved (or not) in the duplex structure. The topographic signal displays a constant periodicity of ~3.1 Myr, according to a Fourier transform calculation. See Supplementary Note 2 for details.

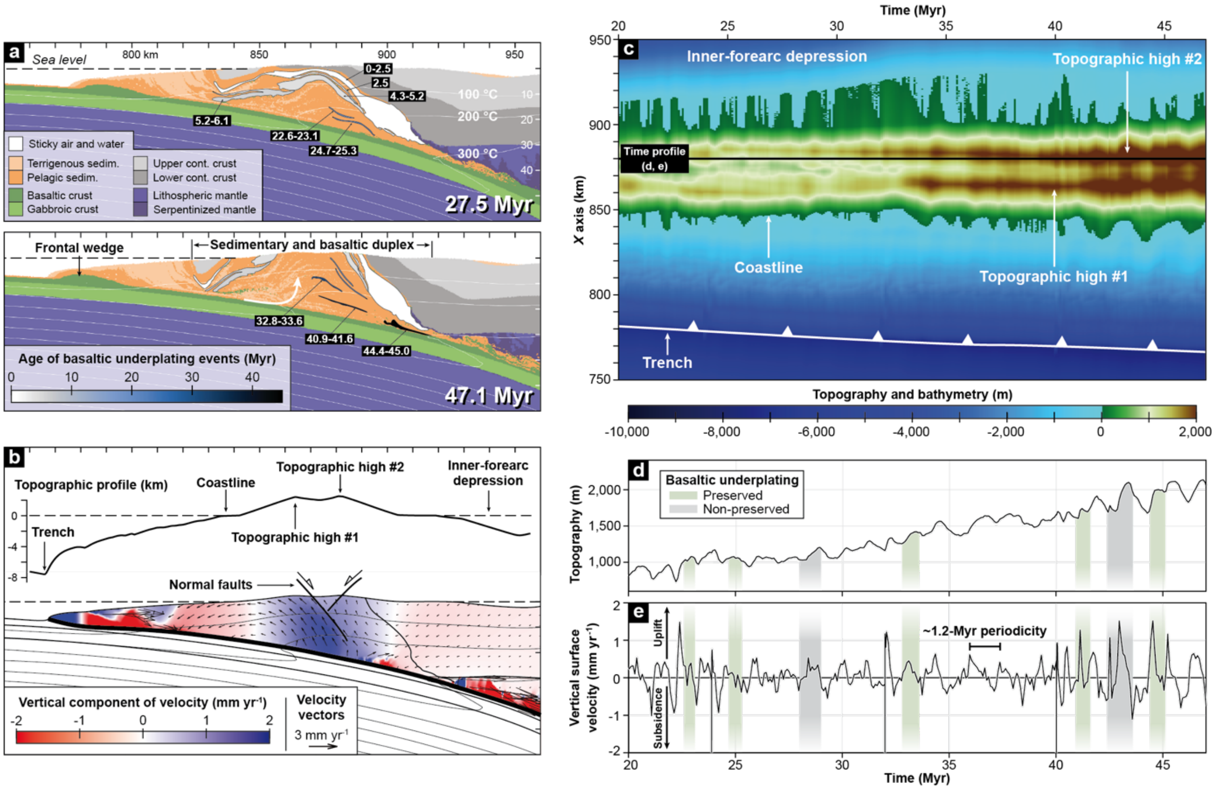

Supplementary Fig. 4 – Tectonic underplating events, duplex formation and forearc topographic evolution for fast-subduction setting (model sed8.0). a. Compositional maps focusing on the forearc domain where tectonic underplating of mostly sedimentary material and minor basaltic crust is achieved by an overall vertical flow (thick white arrow). Once inserted in the duplex, the basaltic slices are highlighted with white-to-blue colours depending on the timing of basal accretion (black boxes; time in Myr). **b.** Velocity map (vertical component only) of the forearc domain during a discrete underplating event. Red and blue colours represent downward and upward vertical displacements, respectively. Black arrows depict the calculated global velocity field. The modelled topographic profile across the forearc domain is depicted with a 2.5 vertical exaggeration. **c.** Dynamics of forearc topography showing the periodic evolution of the coastal topography with alternating uplift and subsidence events and a horizontal migration of the coastline. **d, e.** Time profiles showing the topography (**d**) and vertical surface velocity (**e**) variations of the coastal topography from 20 to 47 Myr (see (**c**) for the exact location of the profile). Green (or grey) bands denote the timing of tectonic underplating of basaltic slices, which are preserved (or not) in the duplex structure. The topographic signal displays a constant periodicity of ~1.2 Myr, according to a Fourier transform calculation. See Supplementary Note 3 for details.

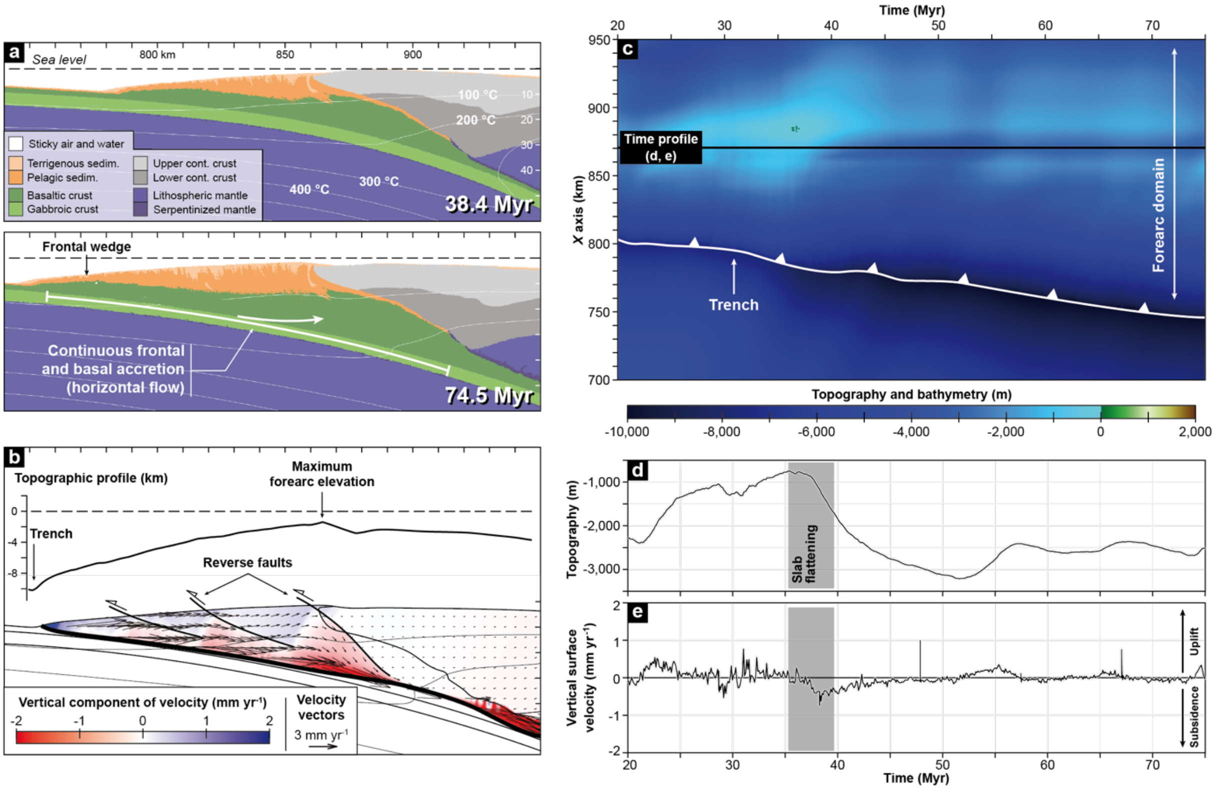

Supplementary Fig. 5 – Tectonic underplating events, accretionary wedge formation and forearc topographic evolution for slow-subduction setting (model sed2.0). a. Compositional maps focusing on the forearc domain where frontal and basal accretion of sedimentary and basaltic material by an overall horizontal flow (thick white arrow) leads for the growth of a wide accretionary wedge. **b.** Velocity map (vertical component only) of the forearc domain. Red and blue colours represent downward and upward vertical displacements, respectively. Black arrows depict the calculated global velocity field. The modelled topographic profile across the forearc domain is depicted with a 2.5 vertical exaggeration. **c.** Dynamics of forearc topography showing a low elevation (i.e., below the sea level) and a non-periodic evolution. **d, e.** Time profiles showing the topography (**d**) and vertical surface velocity (**e**) variations in the forearc domain from 20 to 75 Myr (see (**c**) for the exact location of the profile). Topographic variations are mostly controlled by the dynamics of the subducting lithosphere, such as slab flattening. See Supplementary Note 3 for details.

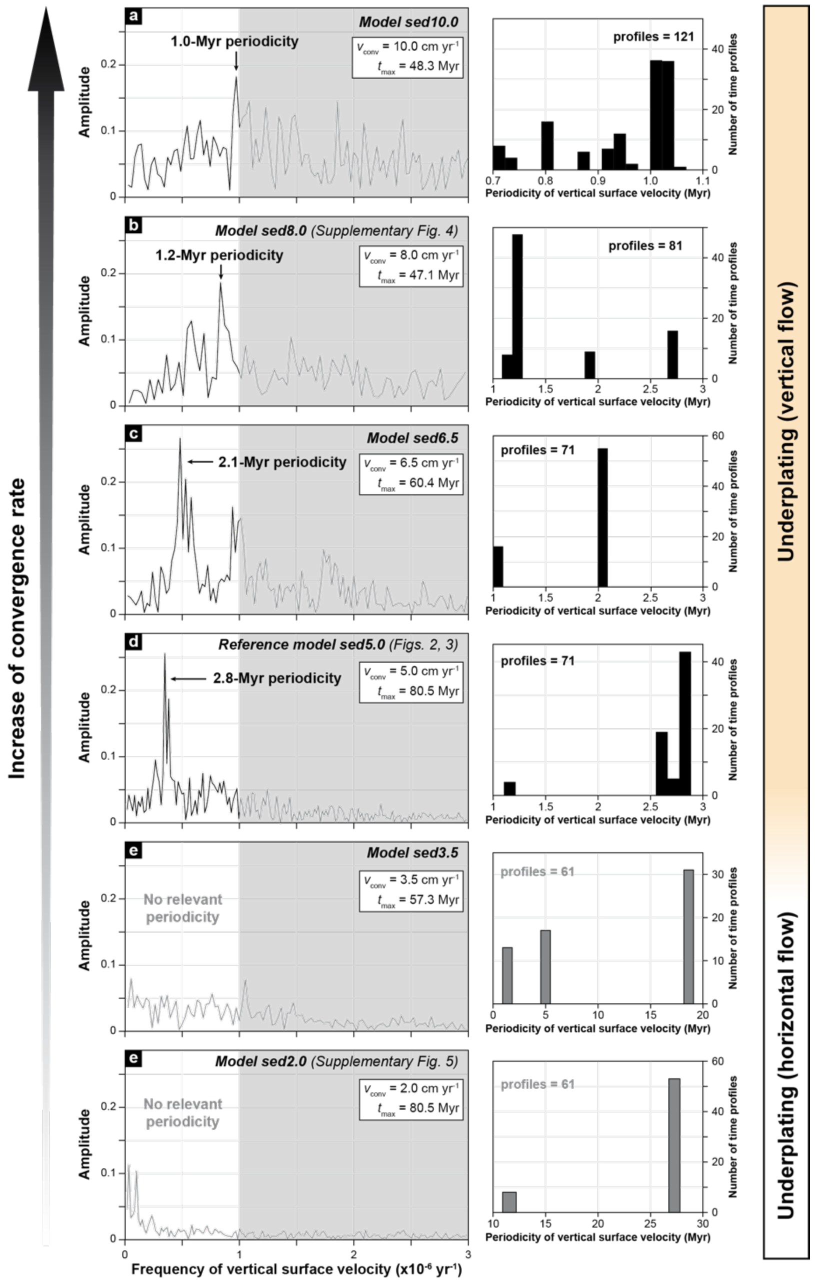

Supplementary Fig. 6 – Compilation of the periodicity of the vertical surface displacements in the forearc domain calculated from numerical experiments with different plate convergence rates. a, b, c, d, e. For each experiment, the periodicity has been obtained by applying a Fourier transform calculation for a set of time profiles across the forearc domain. Left panels show a typical Fourier transform chart for each experiment. Dominant frequency (*f*) is pointed out and labelled with the corresponding periodicity (*P*) with $P = 1/f$. The grey-shaded area depicts $f > 1\text{x}10^{-6}$ yr^{-1} (i.e., $P < 1$ Myr), which is considered as a non-relevant signal because of the minimum ~100-kyr time step in our experiments. V_{conv} and t_{max} indicate the plate convergence velocity and the duration of each experiment, respectively. Right panels show bar diagrams evidencing the distribution of *P* for the complete set of time profiles in each experiment. Note that for slow-subduction models (i.e., models sed3.5 and sed2.0), no relevant periodicity is predicted because of the dominance of a horizontal mass flow at the base of the forearc domain. See Supplementary Note 3 for details.

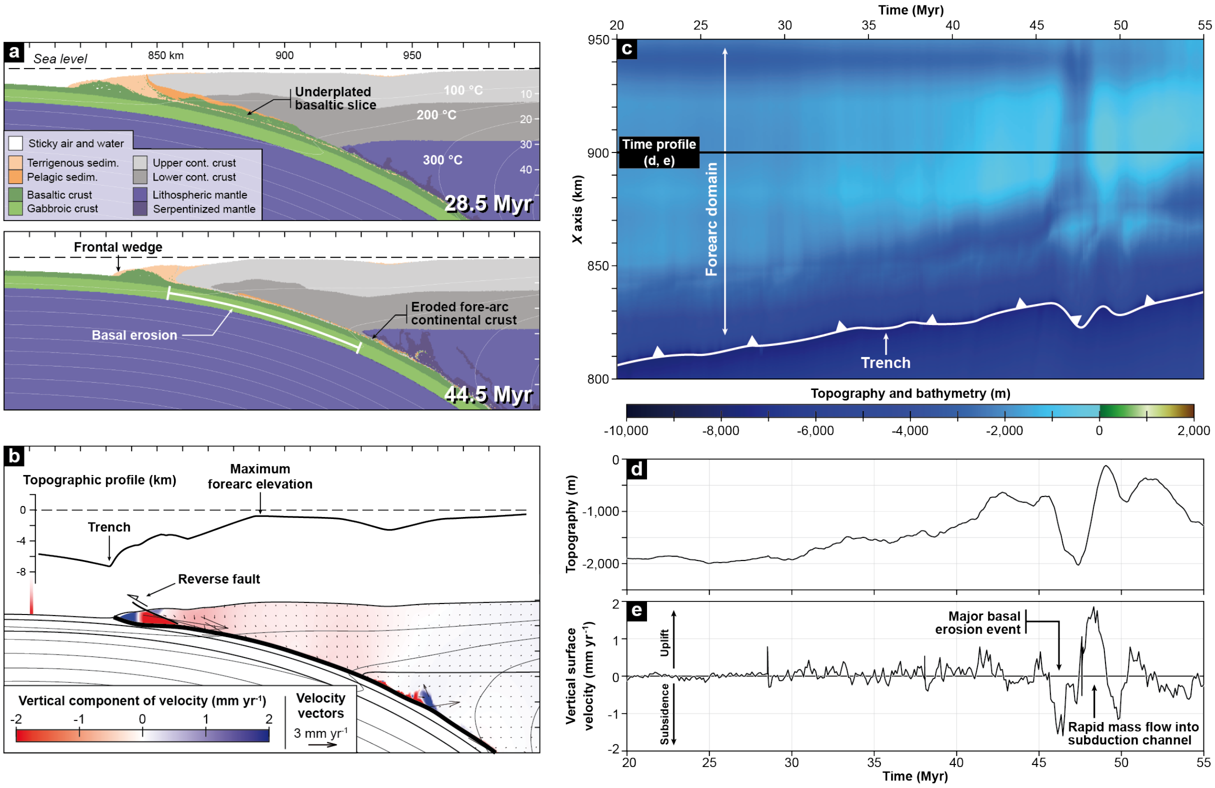

Supplementary Fig. 7 – Basal erosion and forearc topographic evolution for a steady-state erosive margin (model nosed5.0). a. Compositional maps focusing on the forearc domain where basal erosion of initially underplated basaltic slice and continental crust results in the progressive retreat of the trench. **b.** Velocity map (vertical component only) of the forearc domain showing the overall subsidence of the forearc domain in response to continuous basal erosion. Red and blue colours represent downward and upward vertical displacements, respectively. Black arrows depict the calculated global velocity field. The modelled topographic profile across the forearc domain is depicted with a 2.5 vertical exaggeration. **c.** Dynamics of forearc topography showing a low elevation (i.e., below the sea level) and a non-periodic evolution. **d, e.** Time profiles showing the topography (**d**) and vertical surface velocity (**e**) variations in the forearc domain from 20 to 55 Myr (see (**c**) for the exact location of the profile). Topographic variations are mostly controlled by transient mass flows along the plate interface. See Supplementary Note 4 for details.

Supplementary Table 1 – Summary of varying model parameters and main results of numerical experiments.

	Varying model parameters		Results				
Model	Plate convergence rate (cm yr^{-1})	Pelagic sediment thickness (m)	Deep accretionary/erosive dynamics	Underplated material	Long-term forearc topography	Periodic vertical topographic signal	Figures
sed5.0 (reference)	5	500	Tectonic underplating by vertical mass flow	Sediments and basaltic crust	High coastal topography, inner-forearc depression	Yes (~2.8 Myr)	Figs. 2, 3 Sup. Fig. 6
sed6.5	**6.5**	500	Tectonic underplating by vertical mass flow	Sediments, minor basaltic crust	High coastal topography, inner-forearc depression	Yes (~2.1 Myr)	Sup. Fig. 6
sed8.0	**8**	500	Tectonic underplating by vertical mass flow	Mostly sediments	High coastal topography, inner-forearc depression	Yes (~1.2 Myr)	Sup. Figs. 4, 6
sed10.0	**10**	500	Tectonic underplating by vertical mass flow	Mostly sediments	High coastal topography, inner-forearc depression	Yes (~1.0 Myr)	Sup. Fig. 6
sed3.5	**3.5**	500	Tectonic underplating by horizontal mass flow	Sediments and basaltic crust	Low forearc topography	No	Sup. Fig. 6
sed2.0	**2**	500	Tectonic underplating by horizontal mass flow	Basaltic crust and sediments	Low forearc topography	No	Sup. Figs. 5, 6
nosed5.0	5	**0**	Basal erosion	No underplating	Low forearc topography	No	Sup. Fig. 7

Supplementary Table 2 – Thermo-mechanical parameters used in numerical experiments. Effective creep viscosity is calculated as $\eta = \dot{\varepsilon}_{\text{II}}^{\frac{1-n}{n}} A_{\text{D}}^{\frac{1}{n}} \exp\left(\frac{E+P\,V}{n\,R\,T}\right)$, where $\dot{\varepsilon}_{\text{II}}$ is the second invariant of the strain rate tensor, P the pressure, T the temperature and other parameters are defined for all rock types by experimentally constrained flow laws from ref[37] except serpentinized mantle, which is from ref[38].

	Ductile rheology					Plastic rheology		Elastic properties
Material	Flow law	Pre-exponential factor A_{D} (Pa^{-n} s^{-1})	Creep exponent n	Activation energy E (J mol^{-1})	Activation volume V (J Pa^{-1} mol^{-1})	Cohesion (Pa)	Internal friction angle sin (φ_{dry})	Shear modulus μ (Pa)
Sediments (pelagic, terrigenous)	Wet quartzite	1.97×10^{17}	2.3	1.54×10^{5}	8.0×10^{-6}	1.0×10^{7}	0.15	1.0×10^{10}
Upper continental crust	Wet quartzite	1.97×10^{17}	2.3	1.54×10^{5}	1.2×10^{-5}	1.0×10^{7}	0.15	2.5×10^{10}
Lower continental crust	Plagioclase An_{75}	4.80×10^{22}	3.2	2.38×10^{5}	8.0×10^{-6}	1.0×10^{7}	0.15	2.5×10^{10}
Basaltic crust	Plagioclase An_{75}	4.80×10^{22}	3.2	2.38×10^{5}	8.0×10^{-6}	1.0×10^{7}	0.65	2.5×10^{10}
Gabbroic crust	Diabase	1.26×10^{24}	3.4	2.60×10^{5}	8.0×10^{-6}	1.0×10^{7}	0.60	2.5×10^{10}
Dry mantle	Dry olivine	3.98×10^{16}	3.5	5.32×10^{5}	8.0×10^{-6}	1.0×10^{7}	0.60	6.7×10^{10}
Hydrated mantle	Wet olivine	5.01×10^{20}	4.0	4.70×10^{5}	8.0×10^{-6}	1.0×10^{7}	0.10	6.7×10^{10}
Serpentinized mantle	Serpentine	3.21×10^{36}	3.8	8.90×10^{3}	3.2×10^{-8}	1.0×10^{7}	0.10	6.7×10^{10}
Weak zone (hydrated mantle)	Wet olivine	5.01×10^{20}	4.0	4.70×10^{5}	8.0×10^{-6}	1.0×10^{7}	0.10	6.7×10^{10}

Supplementary Table 2 *(continued)*.

	Density calculation			Heat conservation equation		
Material	Density ρ_0 (kg m^{-3})	Thermal expansion α (K^{-1})	Compressibility β (Pa^{-1})	Isobaric heat capacity C_p (J kg^{-1} K^{-1})	Thermal conductivity k (W m^{-1} K^{-1})	Radiogenic heat production H_r (W kg^{-1})
Sediments (pelagic, terrigenous)	2600	3.0×10^{-5}	1.0×10^{-11}	1.0×10^{3}	[0.64+807/(T+77)] exp (4*P*)	2.0×10^{-6}
Upper continental crust	2700	3.0×10^{-5}	1.0×10^{-11}	1.0×10^{3}	[0.64+807/(T+77)] exp (4*P*)	1.0×10^{-6}
Lower continental crust	2950	3.0×10^{-5}	1.0×10^{-11}	1.0×10^{3}	[1.18+474/(T+77)] exp (4*P*)	1.0×10^{-6}
Basaltic crust	3000	3.0×10^{-5}	1.0×10^{-11}	1.0×10^{3}	[1.18+474/(T+77)] exp (4*P*)	2.5×10^{-7}
Gabbroic crust	3000	3.0×10^{-5}	1.0×10^{-11}	1.0×10^{3}	[1.18+474/(T+77)] exp (4*P*)	2.5×10^{-7}
Dry mantle	3200	3.0×10^{-5}	1.0×10^{-11}	1.0×10^{3}	[0.73+1293/(T+77)] exp (4*P*)	2.2×10^{-8}
Hydrated mantle	3200	3.0×10^{-5}	1.0×10^{-11}	1.0×10^{3}	[0.73+1293/(T+77)] exp (4*P*)	2.2×10^{-8}
Serpentinized mantle	3000	3.0×10^{-5}	1.0×10^{-11}	1.0×10^{3}	[0.73+1293/(T+77)] exp (4*P*)	2.2×10^{-8}
Weak zone (hydrated mantle)	3200	3.0×10^{-5}	1.0×10^{-11}	1.0×10^{3}	[0.73+1293/(T+77)] exp (4*P*)	2.2×10^{-8}

Supplementary References

1. Syracuse, E. M., van Keken, P. E. & Abers, G. A. The global range of subduction zone thermal models. *Phys. Earth Planet. Inter.* **183**, 73–90 (2010).
2. Heuret, A. & Lallemand, S. Plate motions, slab dynamics and back-arc deformation. *Phys. Earth Planet. Inter.* **149**, 31–51 (2005).
3. Clift, P. D. & Vannucchi, P. Controls on tectonic accretion versus erosion in subduction zones: Implications for the origin and recycling of the continental crust. *Rev. Geophys.* **42**, (2004).
4. von Huene, R. & Scholl, D. W. Observations at convergent margins concerning sediment subduction, subduction erosion, and the growth of continental crust. *Rev. Geophys.* **29**, 279 (1991).
5. Bassett, D. *et al.* Three-dimensional velocity structure of the northern Hikurangi margin, Raukumara, New Zealand: Implications for the growth of continental crust by subduction erosion and tectonic underplating. *Geochem. Geophys. Geosyst.* **11**, (2010).
6. Henrys, S. *et al.* SAHKE geophysical transect reveals crustal and subduction zone structure at the southern Hikurangi margin, New Zealand. *Geochem. Geophys. Geosyst.* **14**, 2063–2083 (2013).
7. Kimura, H., Takeda, T., Obara, K. & Kasahara, K. Seismic Evidence for Active Underplating Below the Megathrust Earthquake Zone in Japan. *Science* **329**, 210–212 (2010).
8. Tsuji, T., Minato, S., Kamei, R., Tsuru, T. & Kimura, G. 3D geometry of a plate boundary fault related to the 2016 Off-Mie earthquake in the Nankai subduction zone, Japan. *Earth Planet. Sci. Lett.* **478**, 234–244 (2017).
9. Agard, P., Yamato, P., Jolivet, L. & Burov, E. Exhumation of oceanic blueschists and eclogites in subduction zones: Timing and mechanisms. *Earth Sci. Rev.* **92**, 53–79 (2009).
10. Brandon, M. T., Roden-Tice, M. K. & Garver, J. I. Late Cenozoic exhumation of the Cascadia accretionary wedge in the Olympic Mountains, northwest Washington State. *Geol. Soc. Am. Bull.* **110**, 985–1009 (1998).
11. Hashimoto, Y. & Kimura, G. Underplating process from melange formation to duplexing: Example from the Cretaceous Shimanto Belt, Kii Peninsula, southwest Japan. *Tectonics* **18**, 92–107 (1999).
12. Sample, J. C. & Fisher, D. M. Duplex accretion and underplating in an ancient accretionary complex, Kodiak Islands, Alaska. *Geology* **14**, 160 (1986).
13. Willner, A. P. Pressure–Temperature Evolution of a Late Palaeozoic Paired Metamorphic Belt in North–Central Chile (34°–35°30′S). *J. Petrol.* **46**, 1805–1833 (2005).
14. Wells, R. E., Blakely, R. J., Sugiyama, Y., Scholl, D. W. & Dinterman, P. A. Basin-centered asperities in great subduction zone earthquakes: A link between slip, subsidence, and subduction erosion? *J. Geophys. Res.: Solid Earth* **108**, (2003).

15. Hasebe, N., Tagami, T. & Nishimura, S. The evidence of along-arc differential uplift of the Shimanto accretionary complex: Fission track thermochronology of the Kumano Acidic Rocks, Southwest Japan. *Tectonophysics* **224**, 327–335 (1993).

16. Hasebe, N. & Tagami, T. Exhumation of an accretionary prism — results from fission track thermochronology of the Shimanto Belt, southwest Japan. *Tectonophysics* **331**, 247–267 (2001).

17. Moore, G. F., Boston, B. B., Strasser, M., Underwood, M. B. & Ratliff, R. A. Evolution of tectono-sedimentary systems in the Kumano Basin, Nankai Trough forearc. *Marine and Petroleum Geology* **67**, 604–616 (2015).

18. Ramirez, S. G., Gulick, S. P. S. & Hayman, N. W. Early sedimentation and deformation in the Kumano forearc basin linked with Nankai accretionary prism evolution, southwest Japan: Kumano forearc basin early evolution. *Geochem. Geophys. Geosyst.* **16**, 1616–1633 (2015).

19. Clendenen, W. S., Fisher, D. & Byrne, T. Cooling and exhumation history of the Kodiak accretionary prism, southwest Alaska. *Spec. Pap. - Geol. Soc. Am.* **371**, 71–88 (2003).

20. Noda, A. & Miyakawa, A. Deposition and Deformation of Modern Accretionary-Type Forearc Basins: Linking Basin Formation and Accretionary Wedge Growth. in *Evolutionary Models of Convergent Margins - Origin of Their Diversity* (ed. Itoh, Y.) (InTech, 2017). doi:10.5772/67559.

21. McNeill, L. C., Goldfinger, C., Kulm, L. D. & Yeats, R. S. Tectonics of the Neogene Cascadia forearc basin: Investigations of a deformed late Miocene unconformity. *Geol. Soc. Am. Bull.* **112**, 1209–1224 (2000).

22. Coudurier-Curveur, A., Lacassin, R. & Armijo, R. Andean growth and monsoon winds drive landscape evolution at SW margin of South America. *Earth Planet. Sci. Lett.* **414**, 87–99 (2015).

23. Evenstar, L. A. *et al.* Geomorphology on geologic timescales: Evolution of the late Cenozoic Pacific paleosurface in Northern Chile and Southern Peru. *Earth-Science Reviews* **171**, 1–27 (2017).

24. Clift, P. D. & Hartley, A. J. Slow rates of subduction erosion and coastal underplating along the Andean margin of Chile and Peru. *Geology* **35**, 503 (2007).

25. Regard, V. *et al.* Renewed uplift of the Central Andes Forearc revealed by coastal evolution during the Quaternary. *Earth Planet. Sci. Lett.* **297**, 199–210 (2010).

26. Hartley, A. J. *et al.* Development of a continental forearc: A Cenozoic example from the Central Andes, northern Chile. *Geology* **28**, 331 (2000).

27. Armijo, R. & Thiele, R. Active faulting in northern Chile: ramp stacking and lateral decoupling along a subduction plate boundary? *Earth Planet. Sci. Lett.* **98**, 40–61 (1990).

28. Victor, P., Sobiesiak, M., Glodny, J., Nielsen, S. N. & Oncken, O. Long-term persistence of subduction earthquake segment boundaries: Evidence from Mejillones Peninsula, northern Chile. *J. Geophys. Res.* **116**, (2011).

29. Encinas, A., Finger, K. L., Buatois, L. A. & Peterson, D. E. Major forearc subsidence and deep-marine Miocene sedimentation in the present Coastal Cordillera and Longitudinal Depression of south-central Chile (38 30'S-41 45'S). *Geol. Soc. Am. Bull.* **124**, 1262–1277 (2012).

30. Melnick, D. & Echtler, H. P. Inversion of forearc basins in south-central Chile caused by rapid glacial age trench fill. *Geology* **34**, 709 (2006).

31. Melnick, D., Bookhagen, B., Strecker, M. R. & Echtler, H. P. Segmentation of megathrust rupture zones from fore-arc deformation patterns over hundreds to millions of years, Arauco peninsula, Chile. *J. Geophys. Res.: Solid Earth* **114**, (2009).

32. Crameri, F. *et al.* A comparison of numerical surface topography calculations in geodynamic modelling: an evaluation of the 'sticky air' method: Modelling topography in geodynamics. *Geophysical Journal International* **189**, 38–54 (2012).

33. Armijo, R., Lacassin, R., Coudurier-Curveur, A. & Carrizo, D. Coupled tectonic evolution of Andean orogeny and global climate. *Earth Sci. Rev.* **143**, 1–35 (2015).

34. Hayes, G. Slab2 - A Comprehensive Subduction Zone Geometry Model. (2018). doi:10.5066/f7pv6jnv

35. Hirose, F., Nakajima, J. & Hasegawa, A. Three-dimensional seismic velocity structure and configuration of the Philippine Sea slab in southwestern Japan estimated by double-difference tomography. *J. Geophys. Res.* **113**, B09315 (2008).

36. Ryan, W. B. F. *et al.* Global Multi-Resolution Topography synthesis. *Geochem Geophys Geosyst* **10,** (2009).

37. Ranalli, G. *Rheology of the Earth*. (1995).

38. Hilairet, N. *et al.* High-Pressure Creep of Serpentine, Interseismic Deformation, and Initiation of Subduction. *Science* **318**, 1910–1913 (2007).
