# Supplementary material for: Transient stripping of subducting slabs controls periodic forearc uplift
Source: Nat Commun. 2020 Apr 14;11:1823. doi: 10.1038/s41467-020-15580-7 (PMC7156703; doi:10.1038/s41467-020-15580-7)
Supplement: Supplementary file 3 — Description of Additional Supplementary Files [file 41467_2020_15580_MOESM3_ESM.pdf]

Description of Additional Supplementary Files

File Name: Supplementary Movie 1
Description: **Evolution of the reference subduction-zone model (model sed5.0) with tectonic underplating of sedimentary and basaltic material through an overall vertical flow.** Colours indicate the rock composition. White lines indicate isotherms from 100 °C to 1,300 °C (200 °C between two isotherms).

File Name: Supplementary Movie 2
Description: **Evolution of the fast-subduction-zone model (model sed8.0) with mostly tectonic underplating of sedimentary material through an overall vertical flow.** Colours indicate the rock composition. White lines indicate isotherms from 100 °C to 1,300 °C (200 °C between two isotherms).

File Name: Supplementary Movie 3
Description: **Evolution of the slow-subduction-zone model (model sed2.0) with horizontal growth of a mafic-dominated wedge by frontal and basal accretion.** Colours indicate the rock composition. White lines indicate isotherms from 100 °C to 1,300 °C (200 °C between two isotherms).

File Name: Supplementary Movie 4
Description: **Evolution of the subduction-zone model with no pelagic sediments (model nosed5.0), showing a dominant basal erosion dynamics.** Colours indicate the rock composition. White lines indicate isotherms from 100 °C to 1,300 °C (200 °C between two isotherms).
